# Supplementary material for: Resistance Loss in Cemented Paste Backfill Pipelines: Effect of Inlet Velocity, Particle Mass Concentration, and Particle Size
Source: Materials (Basel). 2022 May 6;15(9):3339. doi: 10.3390/ma15093339 (PMC9102405; doi:10.3390/ma15093339)
Supplement: Supplementary file 1 [file materials-15-03339-s001.zip › materials-1681412-supplementary.pdf]

Supplementary materials

Resistance loss in cemented paste backfill pipelines: effect of inlet velocity, viscosity, and particle size

Qiusong Chen [1,2], Hailong Zhou [1], Yunmin Wang [1,2], Xiaoshuang Li [3], Qinli Zhang [1], Yan Feng [1,*] and Chongchong Qi [4,]

1 School of Resources and Safety Engineering, Central South University, Changsha 410083, China; qiusong.chen@csu.edu.cn (Q.C.); 205512082@csu.edu.cn (H.Z.); wangyunmin@csu.edu.cn; zhangqinlicn@126.com (Q.Z.)
2 Sinosteel Maanshan General Institute of Mining Research Co., Ltd, Maanshan 243000, China
3 School of Civil Engineering, Shaoxing University, Shaoxing 312000, China; xsli2011@126.com
4 State Key Laboratory of Safety and Health for Metal Mines, Maanshan 243000, China
* Correspondence: yan.feng@csu.edu.cn

Table S1. Resistance losses at the bend and outlet pipe.

(IV) （m/s）	PMC	PZ （um）	P1(Pa)	P2(Pa)	RL of bend (MPa/km)	RL of the outlet pipe (MPa/km)
1.5	68%	150	7071.8	6723.6	0.492602233	0.33618
		500	5530.8	5222.9	0.435589395	0.261145
		700	3675.4	3421.2	0.359619436	0.17106
		1000	863.71	667.32	0.277835016	0.033366
	70%	150	7445.2	7080.1	0.516510842	0.354005
		500	6138	5804.5	0.471805987	0.290225
		700	4300.6	4024.5	0.390601598	0.201225
		1000	1356.7	1140.6	0.305718962	0.05703
	72%	150	7880.7	7495.8	0.544522112	0.37479
		500	6818.5	6455.8	0.513115537	0.32279
		700	5074.3	4772.8	0.426535247	0.23864
		1000	2108.8	1867.3	0.341652611	0.093365
	74%	150	8411	8002.2	0.578333695	0.40011
		500	7584.9	7189.7	0.559093631	0.359485
		700	5957.5	5624.3	0.471381574	0.281215
		1000	3049.5	2773.1	0.391026011	0.138655
2.0	68%	150	11583	11005	0.81770273	0.55025
		500	9995.9	9439.3	0.787427923	0.471965
		700	7634.6	7167.5	0.660811324	0.358375
		1000	3906.5	3520.8	0.54565388	0.17604
	70%	150	12177	11572	0.855899916	0.5786
		500	10943	10349	0.8403381	0.51745
		700	8644.3	8139.8	0.7137215	0.40699
		1000	4871.4	4449.5	0.596866404	0.222475
	72%	150	12871	12234	0.901170656	0.6117
		500	11979	11343	0.899755945	0.56715
		700	9830.5	9279.1	0.780071428	0.463955
		1000	6104.3	5643.4	0.652040118	0.28217
	74%	150	13701	13025	0.956344369	0.65125
		500	13110	12427	0.966247343	0.62135
		700	11167	10562	0.855899916	0.5281
		1000	7579.4	7071.1	0.719097401	0.353555
2.5	68%	150	17112	16251	1.218065831	0.81255
		500	15572	14712	1.216651121	0.7356
		700	12815	12040	1.096400719	0.602
		1000	8332.4	7700	0.894662987	0.385
	70%	150	17972	17072	1.273239545	0.8536
		500	16841	15929	1.290216072	0.79645
		700	14273	13440	1.178453934	0.672
		1000	9789.2	9105.3	0.967520583	0.455265
	72%	150	18974	18027	1.339730943	0.90135
		500	18242	17272	1.372269287	0.8636
		700	15949	15049	1.273239545	0.75245
		1000	11575	10829	1.055374112	0.54145
	74%	150	20168	19165	1.418954737	0.95825

		500	19854	18819	1.464225476	0.94095
		700	17799	16827	1.375098708	0.84135
		1000	13606	12791	1.152989143	0.63955
3.0	68%	150	23992	22795	1.693408594	1.13975
		500	22131	20921	1.711799832	1.04605
		700	19194	18043	1.628331907	0.90215
		1000	13872	12916	1.352463339	0.6458
	70%	150	24777	23528	1.766973546	1.1764
		500	23788	22504	1.816488417	1.1252
		700	21114	19881	1.744338176	0.99405
		1000	15852	14831	1.444419528	0.74155
	72%	150	26596	25276	1.867417999	1.2638
		500	26727	25211	2.144701278	1.26055
		700	24619	23213	1.989083111	1.16065
		1000	19430	18185	1.761314704	0.90925
	74%	150	27744	26357	1.962203609	1.31785
		500	27728	26275	2.055574509	1.31375
		700	25613	24205	1.991912532	1.21025
		1000	20981	19761	1.725946938	0.98805

Table S2. orthogonal analysis.

**Sample**	**Factors**			
	**IV (m/s)**	**PMC (%)**	**PS (um)**	**Resistance loss (MPa/km)**
1	1.5	68	150	0.336180
2	1.5	70	500	0.290225
3	1.5	72	700	0.238640
4	1.5	74	1000	0.138655
5	2.0	68	500	0.471965
6	2.0	70	700	0.40699
7	2.0	72	1000	0.282170
8	2.0	74	150	0.651250
9	2.5	68	700	0.602000
10	2.5	70	1000	0.455265
11	2.5	72	150	0.901350
12	2.5	74	500	0.940950
13	3.0	68	1000	0.645800
14	3.0	70	150	1.176400
15	3.0	72	500	1.260550
16	3.0	74	700	1.210250
K1	0.250925	0.51398625	0.7662950	-
K2	0.45309375	0.5822200	0.7409225	-
K3	0.72489125	0.6706775	0.6144700	-
K4	1.0732500	0.7352763	0.3804725	-
Range	0.822325	0.2212900	0.3858225	-
Sensitivity ranking	1	3	2	-

Note: K1, K2, K3, K4 : Mean of gradients for each study factor.
